# Supplementary material for: Antimicrobial Activity of Ligilactobacillus animalis SWLA-1 and Its Cell-Free Supernatant against Multidrug-Resistant Bacteria and Its Potential Use as an Alternative to Antimicrobial Agents
Source: Microorganisms. 2023 Jan 11;11(1):182. doi: 10.3390/microorganisms11010182 (PMC9865548; doi:10.3390/microorganisms11010182)
Supplement: Supplementary file 1 [file microorganisms-11-00182-s001.zip › Table S1. AST of indicator bacteria.pdf]

Supplementary Materials

Supplementary Table S1. Antimicrobial susceptbility and resistance of indicator bacteria

	CIP	NAL	IMI	COL	AMP	TET	CHL	AZI	GEN	STR	AMI	SXT	FOT	AXO	FOX	TAZ
Salmonella Gallinarum CNHJ001	R[a]	R	S[b]	R	R	I[c]	S	S	R	R	S	S	S	S	S	S
Salmonella Enteritidis 190610_1	I	R	S	R	R	R	S	S	R	S	S	S	R	R	S	R
Escherichia coli ROH_0034	R	R	R	S	R	R	R	I	S	R	S	R	R	R	R	R
Staphylococcus aureus ROH_0029	I	R	S	R	R	S	S	R	S	S	S	S	R	R	R	R
Escherichia coli ATCC25922	S	S	S	S	S	S	S	S	S	S	S	S	S	S	S	S

Sixteen antimicrobial agents were tested in the Sensititre™ panel to evaluate the antimicrobial susceptibility of *Salmonella* Gallinarum CNHJ001, *Salmonella* Enteritidis 190610_1, *Escherichia coli* ROH_0034, *Staphylococcus aureus* ROH_0029. *Escherichia coli* ATCC25922 was also tested as a susceptible control. The antimicrobial agents used in these experiments were; ciprofloxacin (**CIP**), nalidixic acid (**NAL**), imipenem (**IMI**), colistin (**COL**), ampicillin (**AMP**), tetracycline (**TET**), chloramphenicol (**CHL**), azithromycin (**AZI**), gentamicin (**GEN**), streptomycin (**STR**), amikacin (**AMI**), trimethoprim/sulfamethoxazole (**SXT**), cefotaxime (**FOT**), ceftriaxone (**AXO**), cefoxitin (**FOX**), ceftazidime (**TAZ**).

a. R=resistant *b.* S=susceptible *c.* I=intermediate.
