# Supplementary material for: Antimicrobial Activity of Ligilactobacillus animalis SWLA-1 and Its Cell-Free Supernatant against Multidrug-Resistant Bacteria and Its Potential Use as an Alternative to Antimicrobial Agents
Source: Microorganisms. 2023 Jan 11;11(1):182. doi: 10.3390/microorganisms11010182 (PMC9865548; doi:10.3390/microorganisms11010182)
Supplement: Supplementary file 1 [file microorganisms-11-00182-s001.zip › Table S2. pH_effect_on_indicator_bac revised.pdf]

Supplementary Materials

Supplementary Table S2. Viable counts of bacteria under various pH conditions at each time point

Bacteria	Time (h)	pH conditions				
		pH 6.8	pH 6.5	pH 6.0	pH 5.5	pH 5.0
Salmonella Gallinarum CNHJ001	0	3.45±0.04	3.45±0.03	3.44±0.03	3.43±0.02	3.44±0.04
	4	4.23±0.14	4.07±0.13	4.05±0.12	4.03±0.20	4.04±0.19
	8	5.42±0.08	5.26±0.05	4.29±0.11*	3.87±0.09*	3.84±0.10*
	24	9.24±0.03	9.08±0.06	6.65±0.03*	3.68±0.08*	3.09±0.21*
Salmonella Enteritidis 190610_1	0	3.55±0.07	3.58±0.04	3.58±0.02	3.60±0.02	3.61±0.02
	4	4.93±0.27	4.99±0.08	3.85±0.06*	3.90±0.10*	3.84±0.07*
	8	6.27±0.03	6.04±0.08	4.64±0.13*	3.91±0.08*	3.85±0.09*
	24	9.25±0.06	9.10±0.05	9.04±0.10	3.84±0.12*	3.60±0.18*
Escherichia coli ROH_0034	0	3.55±0.04	3.57±0.02	3.57±0.03	3.56±0.04	3.52±0.04
	4	4.36±0.29	4.40±0.14	4.01±0.17	3.91±0.09*	3.64±0.47*
	8	7.11±0.05	7.20±0.07	5.09±0.10*	3.81±0.09*	3.87±0.04*
	24	9.16±0.05	9.21±0.07	8.48±0.04	3.82±0.07*	3.58±0.13*
Staphylococcus aureus ROH_0029	0	3.49±0.04	3.45±0.03	3.48±0.03	3.48±0.06	3.49±0.03
	4	4.42±0.08	4.43±0.12	4.40±0.14	4.23±0.14	4.17±0.09
	8	8.11±0.05	8.12±0.10	5.09±0.10*	3.77±0.12*	3.86±0.04*
	24	9.16±0.05	9.10±0.16	8.43±0.14	3.77±0.08*	3.58±0.15*

Time-dependent effect of different pH concentrations (pH = 6.8, 6.5, 6.0, 5.5, and 5.0) on bacterial growth in modified TSB-MRS broth. The CFU values (log_{10} CFU/mL) of each indicator bacterium were compared among different pH conditions. Significant ($p < 0.05$) differences between the CFU values at each pH condition are denoted by an asterisk*.
